# Expanding border space improve both yield and stability in a high-density drip-irrigated spring wheat system in Xinjiang, China

**DOI:** 10.3389/fpls.2025.1655063

**Published:** 2025-10-21

**Authors:** Xiaofang Li, Yanhui Zhao, Bianhong Ding, Haoyu Ding, Zhizhong Qiu, Xiaoqi Ma, Yonghui Yang, Wenliang Wan

**Affiliations:** ^1^ The Key Laboratory of Oasis Eco-agriculture, Xinjiang Production and Construction Corps, Shihezi University, Shihezi, China; ^2^ State Key Laboratory of North China Crop Improvement and Regulation, Hebei Agricultural University, Baoding, China; ^3^ Agricultural Science Research Institute of the Sixth Division of Xinjiang Production and Construction Corps, Wujiaqu, China

**Keywords:** drip irrigation, spring wheat, planting pattern, density, productivity

## Abstract

Increasing planting density is regarded as one of the most effective strategies for enhancing wheat productivity. However, it also increases the risk of yield reduction owing to lodging. Preliminary findings suggest that border effects can improve lodging resistance in drip-irrigated wheat systems. Therefore, to maximize grain yield and lodging resistance, we modified the normal drilling sowing (P1, set as CK1) by expanding the border space (EBS) to 20 cm and obtained the corresponding EBS drilling sowing pattern of P2 (drilling sowing, EBS to 20 cm). We also modified the normal uniform sowing pattern (P3, set as CK2) by EBS to 20 cm and obtained the corresponding EBS uniform sowing pattern of P4 (uniform sowing, EBS to 20 cm). A two-year field experiment was conducted to evaluate the yield performance of four sowing patterns (P1–P4) across four planting densities: 570 × 10^4^ plants ha^−1^ (D1), 630 × 10^4^ plants ha^−1^ (D2), 690 × 10^4^ plants ha^−1^ (D3), and 750 × 10^4^ plants ha^−1^ (D4). In both years, the grain yield for all patterns initially increased with planting density and then declined. Compared to P1 and P3, the EBS patterns (P2 and P4) exhibited improved tolerance to high planting densities. Among the tested treatments, P4 pattern achieved the highest grain yield (8576–8779 kg ha^−1^), water use efficiency (15.5–16.0 kg ha^−1^ mm^−1^), and economic return (1991–2058 US$ ha^−1^) at D3. EBS enhanced canopy photosynthesis, optimizing the mobilization of pre-anthesis assimilates stored in vegetative organs toward grains during the filling stage. This redistribution mechanism sustained a high grain weight and spike number under high-density conditions. Furthermore, the improved photosynthetic capacity enhanced stem strength, thereby reducing lodging risk and improving yield stability. Additionally, uniform sowing promoted synchronous development of wheat spikes and reduced harvest losses. Overall, P4 was recommended for high-density drip-irrigated spring wheat systems because of its superior yield performance, stability, water use efficiency, and economic benefits.

## 1 Introduction

Increasing density is key to further enhancing the yield per unit area of crops in Xinjiang but significantly increases the risk of lodging. As one of the world’s most important staple crops, wheat contributes over 20% of the global caloric and protein intake and more than 30% of the human energy supply (Wan et al., 2022a). By 2050, food production should increase by 70% to meet the global demand (Curtis and Halford, 2014). This substantial gap necessitates improvements in crop yield to maintain food security. Current, agronomists have increasingly recognized increasing the number of wheat ears per unit area as the most direct and effective strategy for wheat yield enhancement in Xinjiang (Sang et al., 2024). However, a higher planting density often results in the reduced accumulation of endogenous compounds in stems, diminished mechanical strength, and an increased lodging index, ultimately compromising yield stability (Xiang et al., 2016; Wang, 2022; Wan et al., 2023).

Although traditional lodging-resistance strategies can play a role in high-density planting, they are insufficient to fully meet the demands of wheat production. An increase in planting density should be accompanied by improved lodging resistance, which is typically pursued through the cultivation of density-tolerant, dwarf-type varieties (Sterling et al., 2003; Berry et al., 2006), the application of growth inhibitors via seed soaking or foliar spraying during appropriate growth stages, and the regulated application of nitrogen combined with phosphorus and potassium fertilizers to control plant height and center of gravity (Loycea et al., 2008; Chen, 2011; Wang et al., 2012; Iqbal and Ashraf, 2013). These approaches aim to achieve a balance between a high yield and yield stability. However, several limitations hinder their effectiveness in drip-irrigated wheat systems in Xinjiang. First, density tolerance requires wheat varieties with multiple complex traits such as lodging resistance, regulated tillering, compact plant type, and high photosynthetic efficiency, most of which are controlled by polygenic mechanisms, making accurate varietal selection highly challenging (Shi et al., 2024). Second, although growth inhibitors enhance wheat stem lodging resistance by modulating endogenous hormone pathways (e.g., gibberellins) (Lyu et al., 2021), Xinjiang’s rapid spring temperature rise and short tillering-to-jointing transition period make the precise timing and dosage of such treatments difficult to control (Lv et al., 2019). Third, while water-fertilizer integration technology offers the precise regulation of water and nutrients, its effectiveness is strongly influenced by soil and environmental variability, posing challenges for on-farm implementation (Wan et al., 2022b). It should not be ignored that the aforementioned measures are accompanied by a reduction in total wheat biomass, such as dwarfing plant type, lowered center of gravity, and shortened basal internodes, which can negatively affect yield formation. Therefore, lodging resistance practices in drip-irrigated wheat production in Xinjiang require further systematic investigation and optimization.

Encouragingly, drip-irrigated systems incorporating expanding border space (EBS) have demonstrated notable improvements in both yield and lodging resistance. Over the past decade, our research has focused on optimizing the planting patterns of drip irrigation spring wheat in Xinjiang. In order to reduce the high input cost of drip tubes under the traditional TR4 pattern (one drip tube serves four rows of wheat at 15 cm spacing, denote as TR4), we initially developed the enlarging lateral space (ELS) patterns by expanding the number of rows per tube to six (TR6) and eight (TR8), without altering the row spacing (**Supplementary Figure S1**) (Lv et al., 2019). Although these ELS patterns reduced tube costs, limited lateral water movement led to insufficient irrigation for rows farthest from the tube, ultimately reducing the wheat yield (Lv et al., 2019). We then reconfigured the ELS patterns (TR6 and TR8) by narrowing the row space (NRS), which left border rows with unmodified, large EBS as patterns of TR6L and TR8L, and with modified EBS patterns of TR6S and TR8S (**Supplementary Figure S1**) (Wan et al., 2022a). Subsequently, we found that the optimized NRS patterns (TR6S, TR6L, TR8S, and TR8L) not only improved both productivity and profit but also remarkably showed strong lodging resistance as compared with the normal row space patterns (TR4, TR6, and TR8) (Wan et al., 2022a, 2023). Further data analysis revealed that EBS was the primary factor enhancing the lodging resistance of NRS patterns (TR6S, TR6L, TR8S, and TR8L) (Wan et al., 2023). Therefore, we believe that EBS can play a significant role in improving lodging resistance of drip-irrigated wheat systems in Xinjiang (Wan et al., 2023).

Further research has revealed that under the conventional planting density (570 × 10^4^ plants ha^−1^), EBS can optimize the ventilation and light transmission conditions of the wheat planting system, increase the height and angle of the flag leaf, and improve the photosynthetic performance. And the improvement in photosynthetic performance can promote the accumulation of endogenous substances (lignin and cellulose) in the internodes at the base of the stem, ultimately enhancing the mechanical properties of the stem for lodging resistance and exerting a lodging resistance effect (Wan et al., 2023). Pre-experimental wheat canopy imaging under dense planting confirmed these benefits (**Supplementary Figure S2**). Then, under the dense planting condition, can EBS also exhibit a better effect in lodging resistance? This is of crucial importance for the future high-yield and stable-yield cultivation of drip-irrigated wheat under dense planting conditions in Xinjiang. Based on this, we hypothesized that EBS could enhance both yield and stability under high-density planting. To verify this, conventional drilling and uniform sowing patterns were modified to include a 20 cm EBS, resulting in EBS drilling and EBS uniform sowing patterns. Two-year field trials (2022 and 2023) were conducted to (i) evaluate the yield and water use efficiency (WUE) performance of different drip-irrigated wheat planting patterns, thereby selecting a suitable drip-irrigated planting pattern for high-density conditions in Xinjiang and (ii) clarify the physiological mechanisms by which drip-irrigated wheat planting patterns achieve increased yield and stability under high-density conditions, further verifying the effectiveness of EBS drip-irrigated systems in enhancing yield and stability under high-density planting conditions.

## 2 Materials and methods

### 2.1 Experimental design

Field experiments were conducted during the spring wheat growing seasons of 2022 (Sown on April 3th, harvested on July 16th) and 2023 (Sown on April 5th, harvested on July 14th.) at the experimental station of Shihezi University (44°21′N, 86°04′E; altitude: 450 m). During the growth period, the total solar radiation was 2246 MJ m^−2^ in 2022 and 2355 MJ m^−2^ in 2023. The cumulative temperatures (≥10°C) reached 2051.0°C·d and 2266.5°C·d, while the total precipitation measured 156.6 and 186.3 mm, respectively. The soil type was sandy, with a total porosity of 46.6%, bulk density of 1.31 g cm^−3^, water-holding capacity of 25.8%, and pH of 7.7. Soil properties included 11.8 g kg^−1^ organic matter, 43.2 mg kg^−1^ available nitrogen, 14.0 mg kg^−1^ Olsen phosphorus, and 292 mg kg^−1^ available potassium.

**Figure 1:**
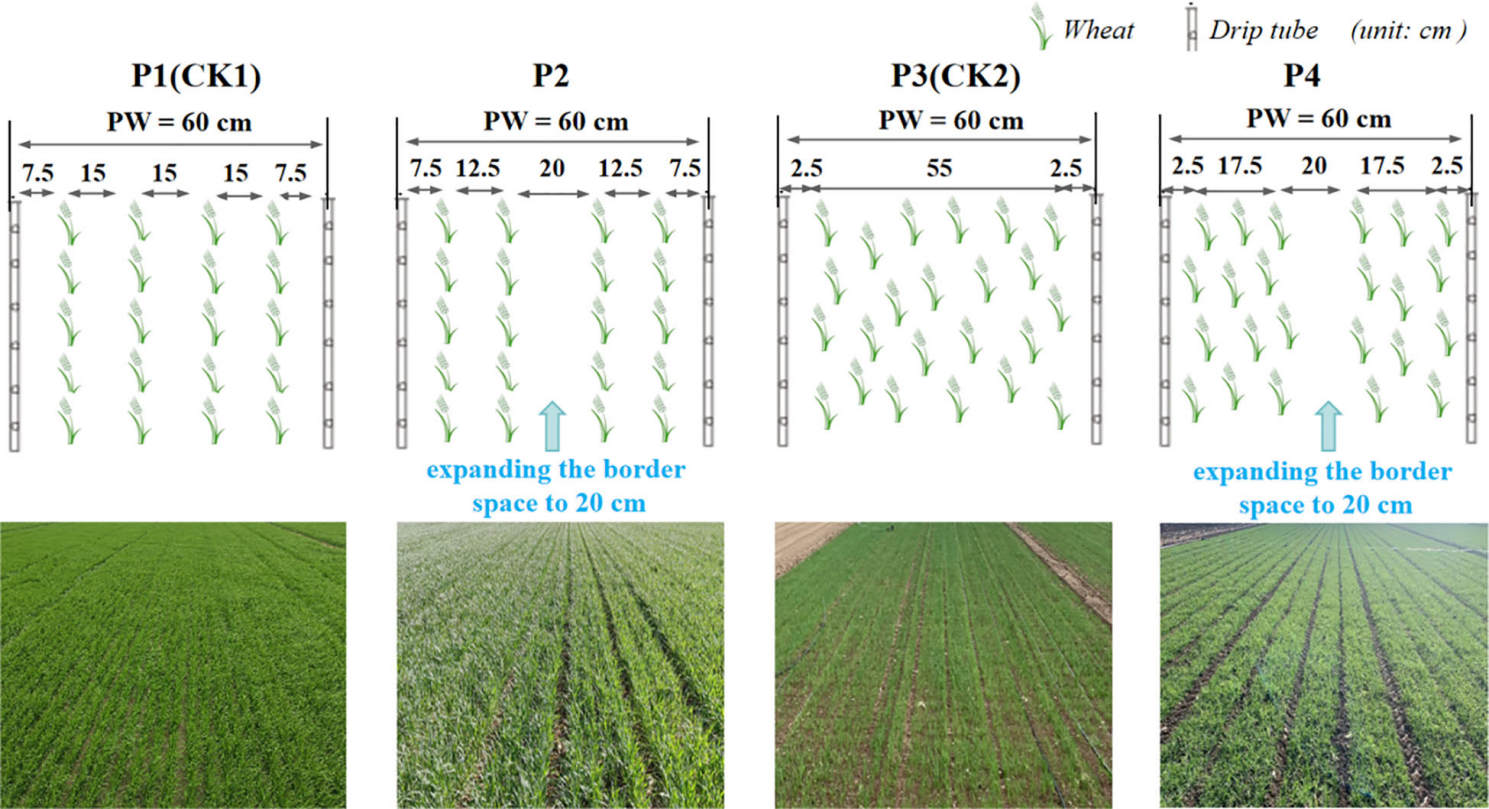
Schematic diagram of four drip irrigation spring wheat planting patterns. P1 indicates the normal drilling planting pattern (set as CK1); P2 indicates the EBS drilling planting pattern; P3 indicates the normal uniform planting pattern (set as CK2); P4 indicates the EBS uniform planting pattern. PW indicates the pattern width (cm).

In this study, the high-yield spring wheat variety Xinchun 44, which is widely cultivated locally, was selected for planting (Wan et al., 2022a). Four drip-irrigated planting patterns were established (**Figure 1**): P1 (normal drilling, a drip irrigation tube was used to supply four rows of wheat, the spacing of adjacent drip irrigation tubes (pattern width) was 60 cm, and the row spacing of wheat was 15 cm, set as CK1); P2 (EBS drilling, a drip irrigation tube was used to supply four rows of wheat, the spacing of adjacent drip irrigation tubes (pattern width) was 60 cm, the row spacing of narrow-row wheat was 12.5 cm, and the row spacing of side-row wheat was widened to 20 cm); P3 (normal uniform, the spacing of adjacent drip irrigation tubes (pattern width) was 60 cm, set as CK2); and P4 (EBS uniform, the spacing of adjacent drip irrigation tubes (pattern width) was 60 cm, and the row spacing of side-row wheat was widened to 20 cm). Four planting densities were set for each planting pattern: D1 (570 × 10^4^ plants ha^−1^), D2 (630 × 10^4^ plants ha^−1^), D3 (690 × 10^4^ plants ha^−1^), and D4 (750 × 10^4^ plants ha^−1^). A split-plot experimental design was used, with planting density as the main plot (secondary factor) and planting pattern as the subplot (primary factor). Each of the 16 treatments was replicated three times, resulting in 48 experimental plots. The experiment utilized specialized precision drilling and uniform sowing planters adapted to different planting patterns (**Supplementary Figure S3**) with drip irrigation tubes simultaneously laid at a shallow depth of 1–2 cm to minimize wind disturbance. To accommodate the sowing width of the planters, each experimental plot exceeded 100 m^2^ in area. Although the rainfall in Xinjiang has increased in recent years (Wan et al., 2022a), due to the large amount of evapotranspiration (about 2000 mm), and the research objective of this experiment does not involve changes in irrigation strategies and fertilization strategies. Therefore, irrigation and fertilization strategies for the two experimental years followed previously published protocols and local agronomic recommendations (Liu et al., 2013; Lv et al., 2019). That is, the irrigation water amounts at the three-leaf, jointing, booting, anthesis, early milk, and late milk stages were 900, 900, 900, 675, 675, and 450 m^3^ ha^−1^, respectively. The corresponding nitrogen application rates at the pre-sowing, three-leaf, jointing, booting, anthesis, and early milk stages were 60, 36, 96, 48, 36, and 24 kg ha^−1^, respectively.

### 2.2 Sampling and measurements

#### 2.2.1 Biomass, spike layer height, and ratio of spikes suitable for harvest

Following the methods of Wheeler et al. (1996) and Plaut et al. (2004), wheat spikes that flowered on the same day and exhibited uniform growth were marked at anthesis for sampling. At both anthesis and maturity stages, 20 plants per treatment were randomly selected from three replicates. At anthesis, the plant samples were separated into leaves, stems, sheaths, and spikes, whereas at maturity, they were divided into leaves, stems, sheaths, glumes, and grains. The samples were initially heat-treated at 105°C for 30 min, followed by drying at 85°C to a constant weight to determine total dry matter accumulation (TDM, kg m^−2^). The dry matter remobilized after anthesis (DMR, kg m^−2^), redistribution rate of DMR to grains (DMR-R, %), contribution rate of DMR to grains (DMR-C, %), and harvest index (HI) were calculated using Equations 1-4 (Wheeler et al., 1996; Plaut et al., 2004; Lv et al., 2019; Wan et al., 2022a):


(1) $$\begin{array}{c} \text{DMR }(\text{kg}\cdot {\text{m}}^{-2})=\text{d}\text{r}\text{y}\;\text{m}\text{a}\text{t}\text{t}\text{e}\text{r}\;\text{a}\text{c}\text{c}\text{u}\text{m}\text{u}\text{l}\text{a}\text{t}\text{i}\text{o}\text{n}\;\text{a}\text{t}\;\text{a}\text{n}\text{t}\text{h}\text{e}\text{s}\text{i}\text{s}\;(\text{k}\text{g}\cdot {\text{m}}^{-2})\\ -\text{d}\text{r}\text{y}\;\text{m}\text{a}\text{t}\text{t}\text{e}\text{r}\;\text{a}\text{c}\text{c}\text{u}\text{m}\text{u}\text{l}\text{a}\text{t}\text{i}\text{o}\text{n}\;\text{o}\text{f}\;\text{v}\text{e}\text{g}\text{e}\text{t}\text{a}\text{t}\text{i}\text{o}\text{n}\;\text{a}\text{t}\;\text{m}\text{a}\text{t}\text{u}\text{r}\text{i}\text{t}\text{y}\;(\text{kg}\cdot {\text{m}}^{-2}) \end{array}$$

(2) $$\begin{array}{c} \text{D}\text{M}\text{R}-\text{R }(\%)=\text{D}\text{M}\text{R}(\text{kg}\cdot {\text{m}}^{-2})\\ /\text{D}\text{r}\text{y}\;\text{m}\text{a}\text{t}\text{t}\text{e}\text{r}\;\text{a}\text{c}\text{c}\text{u}\text{m}\text{u}\text{l}\text{a}\text{t}\text{i}\text{o}\text{n}\;\text{a}\text{t}\;\text{a}\text{n}\text{t}\text{h}\text{e}\text{s}\text{i}\text{s}\;(\text{kg}\cdot {\text{m}}^{-2})\times 100\% \end{array}$$

(3) $$\begin{array}{c} \text{DMR}-\text{C }(\%)=\text{D}\text{M}\text{R}(\text{kg}\cdot {\text{m}}^{-2})\\ /\text{G}\text{r}\text{a}\text{i}\text{n}\;\text{w}\text{e}\text{i}\text{g}\text{h}\text{t}\;\text{a}\text{t}\;\text{m}\text{a}\text{t}\text{u}\text{r}\text{i}\text{t}\text{y}\;(\text{kg}\cdot {\text{m}}^{-2})\times 100\% \end{array}$$

(4) $$\begin{array}{c} \text{HI}=\text{G}\text{r}\text{a}\text{i}\text{n}\;\text{w}\text{e}\text{i}\text{g}\text{h}\text{t}\;(\text{k}\text{g}\cdot {\text{m}}^{-2})\\ /\text{D}\text{r}\text{y}\;\text{m}\text{a}\text{t}\text{t}\text{e}\text{r}\;\text{a}\text{c}\text{c}\text{u}\text{m}\text{u}\text{l}\text{a}\text{t}\text{i}\text{o}\text{n}\;\text{a}\text{t}\;\text{m}\text{a}\text{t}\text{u}\text{r}\text{i}\text{t}\text{y}\;(\text{k}\text{g}\cdot {\text{m}}^{-2}) \end{array}$$

Around July 15th, during the wheat wax ripeness stage, 30 plants were randomly selected under each drip-irrigated pattern to measure the distance from the middle of the spike to the ground (cm), and the average value was recorded as the spike layer height. Simultaneously, the spikes were harvested and threshed, and grain moisture content was determined using the drying method. In line with local post-harvest practices in Xinjiang, where wheat was typically transported directly to grain stations without sun-drying, a grain moisture content ≤13% was considered suitable for harvest (fully filled), while values >13% were classified as unsuitable (incompletely filled) (Niu et al., 2024). The ratio of spikes suitable for harvest (%) was calculated as the percentage of fully filled spikes among the 30 sampled plants.

#### 2.2.2 Evapotranspiration and water use efficiency

ET_c_ was calculated using Equation 5 (Lv et al., 2019; Wan et al., 2022b):


(5) $${\text{ET}}_{\text{c}}=\text{I}+\text{P}+{\text{C}}_{\text{r}}-{\text{R}}_{\text{f}}-{\text{D}}_{\text{p}}\pm \text{ΔS}$$

ET_c_ refers to the total evapotranspiration (mm) of wheat throughout the growth period. Parameter I denotes the cumulative irrigation amount (mm), and P represents the total precipitation (mm), which can be measured using a compact meteorological device installed at the experimental site. The parameters C_r_, D_p_, and R_f_ correspond to the capillary rise, deep percolation, and surface runoff, respectively. Because no significant rainfall occurred during the study, no surface runoff was observed. Owing to the low groundwater level at the site, C_r_, R_f_, and D_p_ were all considered zero (The soil volume moisture content in the 0–40 cm layer of the drip-irrigated wheat fields in Xinjiang was only approximately 20% before irrigation. Therefore, C_r_ was almost zero. Secondly, the groundwater level in Xinjiang is relatively deep, and there is no recharge from groundwater. Thus, D_p_ was usually also zero. Moreover, due to the dry soil, surface runoff does not occur under drip irrigation conditions, so R_f_ was also zero. The calculation method of this experiment was consistent with that of previous studies (Lv et al., 2019)). The change in soil moisture before sowing and after harvest (ΔS, mm) was determined using the soil auger (oven drying) method. Water use efficiency (WUE) was calculated as the ratio of grain yield (kg ha^−1^) to ET_c_ (mm) (Du et al., 2008; Payero et al., 2008; Kresovi´c et al., 2016).

#### 2.2.3 Photosynthetic capacity, stem strength, and lodging rate

During the grain filling stage, photosynthetic performance and stem strength were assessed by selecting 10 uniformly growing wheat plants from each of the three replicates per treatment. The net photosynthetic rate of the flag leaf was measured using a portable photosynthesis system (LI-6400; LI-COR Inc., NE, USA) under a light intensity of 1700 μmol CO_2_ mol^−1^. The stem bending strength was determined following the method described by Peng et al. (2014) using a stem strength tester (YYD-1, Hangzhou TOP Instrument Co., Ltd., China). Specifically, the second internode (with sheath) from ten randomly selected plants per replicate was positioned on a support column with a 5 cm gap. A perpendicular force was applied via the SBS probe until the internode bent or broke. The maximum force applied at the point of failure was recorded as stem bending strength (N). The lodging rate for each planting pattern was calculated using Equation 6:


(6) $$\begin{array}{c} \text{Lodging rate }(\%)\\ =\text{Number of wheat plants that lodged}/\text{Total number of plants}\\ \times 100\% \end{array}$$

A plant was classified as lodged when the angle between the stem and ground was less than or equal to 45° (Keller et al., 1999). The lodging rate was determined by surveying wheat within a 0.6 m^2^ area (0.6 m width between adjacent drip tubes × 1 m length).

#### 2.2.4 Yield, cost, and benefit

The yield and its components, including spike numbers, grains per spike, and 1000-grain weight, were measured following the method described by Lv et al. (2019). At maturity, the number of spikes per plot was recorded, and 30 plants were sampled for indoor analysis of grains per spike and 1000-grain weight. Subsequently, a 1 m × 0.6 m area of wheat was harvested from each plot to determine grain yield (kg ha^−1^). The economic profit was calculated using Equation 7.


(7) $$\begin{array}{c} \text{Economic profit }(\text{US}\${\text{ ha}}^{-1})=\text{overall grain yield }({\text{kg ha}}^{-1})\\ \times \text{wheat price }(\text{US}\${\text{ kg}}^{-1})-\text{irrigation equipment cost }(\text{US}\${\text{ ha}}^{-1})\\ -\text{fertilizer cost }(\text{US}\${\text{ ha}}^{-1})-\text{pesticide cost }(\text{US}\${\text{ ha}}^{-1})\\ -\text{seed cost }(\text{US}\${\text{ ha}}^{-1})-\text{water cost }({\text{m}}^{3}{\text{ha}}^{-1}) \end{array}$$

### 2.3 Statistical analysis

All data were analyzed using one-way analysis of variance (ANOVA) in SPSS (SPSS Inc., Chicago, IL, USA) to assess differences among planting patterns (Before conducting the ANOVA analysis, we performed the normality test and the homogeneity of variance test, and used LSR (Least Significant Difference) to determine the significant differences among the mean values of different treatment groups (that is, when *P* < 0.05, it indicates that there is a significant difference among the mean values of different treatment groups; when *P* ≥ 0.05, it indicates that there is no significant difference among the mean values of different treatment groups). Figures were produced using Origin 2021 (Systat Software, Inc., San Jose, CA, USA).

## 3 Results and discussion

### 3.1 Expanding border space uniform sowing pattern can effectively increase yield and WUE under a high-density drip-irrigated system

At a traditional planting density of 570 × 10^4^ plants ha^−1^ (D1), the wheat grain yields of the normal patterns P1 (CK1) and P3 (CK2) ranged from 7902 to 8246 and 8177 to 8365 kg ha^−1^, respectively (**Table 1**). When the planting density was increased to 630 × 10^4^ plants ha^−1^ (D2), the grain yield for all planting patterns (P1, P2, P3, and P4) increased to some extent. Among them, the grain yields of normal pattern (P1 and P3) were 7992–8367 kg ha^−1^ and 8365–8565 kg ha^−1^, respectively. In most cases, the grain yield of the EBS patterns (P2 and P4) was only slightly higher than that of the corresponding normal patterns (P1 and P3) (There was no significant difference between the normal pattern and their corresponding EBS pattern, except that the yield of P2 was significantly higher than that of P1 in 2023). Interestingly, at a higher density of 690 × 10^4^ plants ha^−1^ (D3), the yields of the normal patterns (P1 and P3) declined, whereas those of the EBS patterns (P2 and P4) continued to increase. This indicates that D3 is the limiting density for further yield increases in the normal patterns (P1 and P3) in this experiment. At this planting density level, the grain yield of the EBS patterns (P2 and P4) was significantly higher than that of the corresponding normal patterns (P1 and P3). When the planting density was further increased to 750 × 10^4^ plants ha^−1^ (D4), the EBS patterns (P2 and P4) also showed a decreasing trend in yield. This indicates that D4 is the limiting density for further yield increases in the EBS pattern (P2 and P4) in this experiment. Notably, under this planting density level, the grain yield of the EBS patterns (P2 and P4) was also significantly higher than that of the corresponding normal patterns (P1 and P3). It is evident that the EBS patterns (P2 and P4) had better resistance to high-density planting, maintaining superior productivity up to a dense planting level of 690 × 10^4^ plants ha^−1^ (D3), with the EBS uniform sowing pattern (P4) showing the highest yield level at D3, reaching 8576–8779 kg ha^−1^.

**Table 1:** Performance of the four planting patterns in terms of yield, evapotranspiration (ET_c_), water use efficiency (WUE), total cost, and profit under different planting density levels.

Year & treatment		Yield		ET_c_		WUE		Total cost		Economic profit	
		Kg ha^-1^	DT_D1P1_ (%)	Mm	DT_D1P1_ (%)	Kg ha^-1^mm^-1^	DT_D1P1_ (%)	US$ha^-1^	DT_D1P1_ (%)	US$ha^-1^	DT_D1P1_ (%)
2022	P1	8246 ^de^	—	570 ^ab^		14.5 ^de^		748.9		1960 ^bc^	
	P2	8225 ^de^	-0.3	576 ^a^	1.1	14.3 ^e^	-1.5	748.9	0.0	1954 ^c^	-0.3
D1	P3	8365 ^cd^	1.4	558 ^d^	-2.1	15.0 ^c^	3.4	748.9	0.0	2000 ^abc^	2.0
	P4	8353^cd^	1.3	563 ^cd^	-1.2	14.8 ^cd^	2.3	748.9	0.0	1996 ^abc^	1.8
D2	P1	8367 ^cd^	1.5	562 ^cd^	-1.4	14.9 ^cd^	2.7	787.9	5.2	1961 ^bc^	0.0
	P2	8456 ^bcd^	2.6	570 ^ab^	0.0	14.8 ^cd^	2.3	787.9	5.2	1991 ^abc^	1.5
	P3	8565 ^abc^	3.9	551 ^ef^	-3.3	15.5 ^b^	7.2	787.9	5.2	2026 ^abc^	3.4
	P4	8605 ^ab^	4.4	557 ^de^	-2.3	15.5 ^b^	6.6	787.9	5.2	2040 ^ab^	4.0
D3	P1	7685 ^gh^	-6.8	556 ^def^	-2.5	13.8 ^f^	-4.7	826.9	10.4	1698 ^e^	-13.4
	P2	8553 ^abc^	3.7	565 ^bc^	-0.9	15.1 ^bc^	4.4	826.9	10.4	1983 ^abc^	1.2
	P3	7987 ^f^	-3.4	543 ^g^	-4.7	14.7 ^cd^	1.4	826.9	10.4	1797 ^d^	-8.3
	P4	8779 ^a^	6.5	550 ^f^	-3.5	16.0 ^a^	10.1	826.9	10.4	2058 ^a^	5.0
D4	P1	7370 ^i^	-10.6	550 ^f^	-3.5	13.4 ^g^	-7.6	865.9	15.6	1556 ^g^	-20.7
	P2	7886 ^fg^	-4.4	558 ^d^	-2.1	14.1 ^ef^	-2.5	865.9	15.6	1725 ^de^	-12.0
	P3	7587 ^h^	-8.0	538 ^g^	-5.6	14.1 ^ef^	-2.8	865.9	15.6	1627 ^f^	-17.0
	P4	8065 ^ef^	-2.2	543 ^g^	-4.7	14.9 ^cd^	2.4	865.9	15.6	1784 ^d^	-9.0
2023	P1	7902 ^d^	—	573 ^bc^		13.8 ^efg^		748.9		1847 ^d^	
	P2	7925 ^d^	0.3	579 ^a^	1.1	13.7 ^fgh^	-0.9	748.9	0.0	1855 ^d^	0.4
D1	P3	8177 ^c^	3.5	560 ^de^	-2.2	14.6 ^b^	5.7	748.9	0.0	1938 ^abc^	4.9
	P4	8201 ^c^	3.8	568 ^c^	-0.9	14.4 ^bc^	4.7	748.9	0.0	1946 ^abc^	5.3
D2	P1	7992 ^d^	1.1	567 ^cd^	-1.1	14.1 ^cde^	2.2	787.9	5.2	1838 ^d^	-0.5
	P2	8256 ^c^	4.5	574 ^ab^	0.2	14.4 ^bcd^	4.2	787.9	5.2	1925 ^bc^	4.2
	P3	8365 ^bc^	5.8	554 ^f^	-3.3	15.1 ^a^	9.4	787.9	5.2	1961 ^abc^	6.1
	P4	8452 ^ab^	7.0	560 ^ef^	-2.3	15.1 ^a^	9.4	787.9	5.2	1989 ^ab^	7.7
D3	P1	7454 ^fg^	-5.7	561 ^de^	-2.2	13.3 ^h^	-3.6	826.9	10.4	1622 ^f^	-12.2
	P2	8353 ^bc^	5.7	568 ^bc^	-0.9	14.7 ^b^	6.6	826.9	10.4	1918 ^c^	3.8
	P3	7659 ^e^	-3.1	547 ^g^	-4.6	14.0 ^def^	1.5	826.9	10.4	1689 ^e^	-8.5
	P4	8576 ^a^	8.6	554 ^f^	-3.3	15.5 ^a^	12.2	826.9	10.4	1991 ^a^	7.8
D4	P1	6995 ^h^	-11.5	554 ^f^	-3.3	12.6 ^i^	-8.5	865.9	15.6	1432 ^h^	-22.5
	P2	7586 ^ef^	-4.0	561 ^de^	-2.1	13.5 ^gh^	-2.1	865.9	15.6	1627 ^f^	-11.9
	P3	7299 ^g^	-7.6	545 ^g^	-4.8	13.4 ^gh^	-3.0	865.9	15.6	1532 ^g^	-17.1
	P4	7653 ^e^	-3.2	543 ^g^	-5.2	14.1 ^cde^	2.2	865.9	15.6	1649 ^ef^	-10.8
*F _Year_*		156.990^**^		91.802^**^		228.249^**^				156.990^**^	
*F _Density_*		21.762^**^		283.148^**^		13.375^**^				11.908^**^	
*F _Pattern_*		10.660^**^		280.778^**^		8.811^**^				10.664^**^	

P1: normal drilling planting pattern; P2: EBS (Expanding Border Space) drilling planting pattern; P3: normal uniform planting pattern; P4: EBS uniform planting pattern. D1, D2, D3, and D4 represent planting densities of 570 × 10^4^, 630 × 10^4^, 690 × 10^4^, and 750 × 10^4^ plants ha^−1^, respectively. DT_D1P1_ indicates the percentage increase or decrease in other treatment combinations relative to the D1P1 treatment. Within the same column, different lowercase letters indicate significant differences between treatments (*P* < 0.05). F_Year_, F_Density_, and F_Pattern_ are the F-values for the year, planting density, and planting patterns, respectively. ** indicates significant differences in F-values at the *P* < 0.01 level.

As planting density increased, ET_c_ showed a gradual decline (**Table 1**). This is understandable, as in Xinjiang, where solar radiation is intense, a denser wheat canopy often helps reduce solar radiation on the soil surface, thereby decreasing soil moisture evaporation and subsequently lowering the calculated ETc values (Wan et al., 2022a; Gao et al., 2021). Therefore, at the same planting densities, EBS patterns (P2 and P4) typically show higher ET_c_ values, indicating that the widened marginal space increases the ability of solar radiation to penetrate the wheat canopy, which may actually increase soil moisture evaporation. Certainly, previous studies have indicated that a higher planting density can intensify intra-species competition, thereby improving root capacity for soil moisture uptake (Gao et al., 2021). This competition of root systems for soil moisture is bound to lead to a result, that is, the water utilization capacity of crops under high-density conditions may be higher. The data from this experiment confirmed this, that is, the data demonstrated that WUE generally increased with planting density (from D1 to D3). In this study, the EBS uniform sowing pattern (P4) exhibited the highest WUE under a dense planting density of 690 × 10^4^ plants ha^−1^ (D3), reaching 15.5–16.0 kg ha^−1^ mm^−1^. Given that irrigation, fertilization, and drip irrigation infrastructure remained consistent across treatments, the cost differences mainly resulted from the seed input. At traditional density, the input cost was 748.9 US$ ha^−1^. With increasing density, the input costs increased by 5.2%, 10.4%, and 15.6% for D2, D3, and D4, respectively. Ultimately, P4 at D3 achieved the highest economic benefit, ranging from 1991 to 2058 US$ ha^−1^.

Furthermore, at the same density, the yield, water use efficiency (WUE), and profit of the normal uniform sowing pattern of P3 (CK2) were generally higher than those of the normal drilling sowing pattern of P1 (CK1). Similarly, the yield, WUE, and profit of the EBS uniform sowing pattern of P4 were also generally higher than those of the EBS drilling sowing pattern of P2. This indicates that under the drip irrigation conditions in Xinjiang, the uniform sowing patterns is actually more suitable for the growth and development of drip-irrigated wheat, and this phenomenon becomes more pronounced as the density increases (In most cases, there are significant differences in yield, WUE, and profit between P1 and P2, P3 and P4 under high-density conditions (D2, D3, D4); while in most cases, there are no significant differences in yield, WUE, and profit between P1 and P2, P3 and P4 under conventional density conditions (D1)). This is indeed understandable, because the even distribution of wheat seeds can effectively reduce the competition among plants for resources, ensure the coordination between individual plants and the plant community, and make the plants grow more robust. This is consistent with the previous research results on uniform sowing (Zhang, 2022).

### 3.2 The EBS patterns effectively mitigated the decreased degree of grain number per spike and grain weight under dense planting conditions

**Figure 2:**
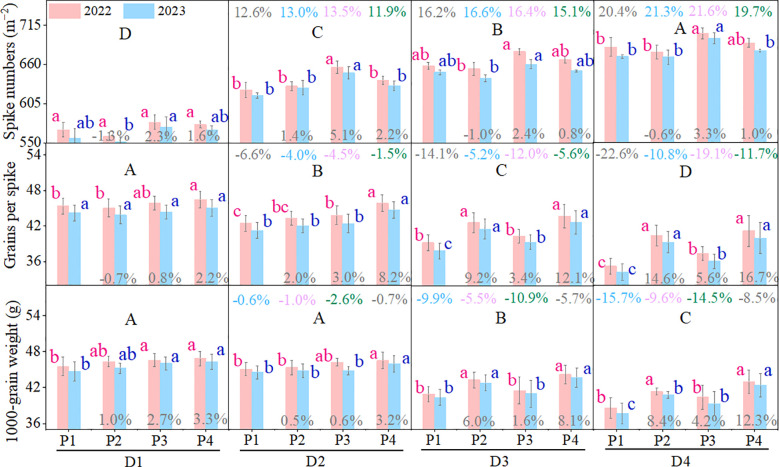
Performance of wheat yield components (spike numbers, grains per spike, and 1000-grain weight) for the four patterns. D1, D2, D3, and D4 represent planting densities of 570 × 10^4^, 630 × 10^4^, 690 × 10^4^, and 750 × 10^4^ plants ha^–1^, respectively. P1: normal drilling pattern; P2: EBS drilling pattern; P3: normal uniform sowing pattern; P4: EBS uniform sowing pattern. Lowercase letters indicate significant differences (*P* < 0.05) in yield components among planting patterns at the same planting density. Uppercase letters indicate significant differences (*P* < 0.05) among planting densities within the same planting pattern. Numbers displayed at the top of each column represent the percentage change in yield components under high-density planting levels (D2, D3, and D4) compared with the conventional density (D1). Numbers at the bottom of each column represent the percentage change in yield components of P2, P3, and P4 relative to P1 under the same planting density. All percentage changes were calculated based on the average values from 2022 and 2023.

In crop production, yield variation under different treatments was pin fact dependent on the changes in yield components (effective spike numbers, grains per spike, and 1000-grain weight) (Sha et al., 2025). In this experiment, the effective spike numbers for all planting patterns (P1, P2, P3, and P4) increased progressively with increasing planting density (compared to D1, when the planting density increased to D2, the increases in effective spike numbers for P1, P2, P3, and P4 were respectively 12.6%, 13.0%, 13.5%, 11.9%; when the planting density increased to D3, the increases for P1, P2, P3, and P4 were respectively 16.2%, 16.6%, 16.4%, 15.1%; when the planting density increased to D4, the increases for P1, P2, P3, and P4 were respectively 20.0%, 21.3%, 21.6%, 19.7%) (**Figure 2**). It is evident that higher planting densities enhance wheat yield primarily by increasing the number of effective spikes per unit area, which is consistent with previous studies (Shao et al., 2024; Liu et al., 2021). It is noteworthy that in most cases, there were no significant differences in the number of effective spikes among the different patterns (P1, P2, P3, and P4) at both traditional planting density (D1) and high planting density (D2, D3, and D4). This indicates that under drip-irrigated systems in Xinjiang, the planting pattern had a limited effect on wheat effective spike numbers. In fact, when the water and fertilizer conditions are the same, the number of effective spikes of wheat mainly depends on its own tillering ability (Xie et al., 2006). However, in Xinjiang, the temperature rises relatively quickly in spring, and this often results in insufficient formation of effective tillers (Lv et al., 2019). Therefore, the final number of spikes in the wheat population in Xinjiang depends on the number of spikes formed on the main stem, which in turn directly depends on the seeding rate. Although the normal uniform pattern of P3 exhibited slightly better performance in effective spike numbers, the increase was not substantial (compared to traditional P1, the increase in effective spike numbers for P3 was only 2.3% to 5.1%) (**Figure 2**). Therefore, in this experiment, the effective spike numbers could not be primarily attributed to the main reason why the EBS uniform sowing pattern (P4) grain yield effectively increased under the high-density drip-irrigated system.

Regarding grains per spike, under the conventional planting density (D1), there was little difference in the number of grains per spike across various planting patterns (only except for the grains per spike of P4, which significantly exceeded other patterns in 2022) (**Figure 2**). Previous studies have shown that owing to the competitive relationship between crops and water, fertilizer, growth space, light, and other resources, increasing planting density can boost the number of groups but concurrently aggravate competition among groups, resulting in a decrease in grain number per spike and 1000-grain weight (Shao et al., 2024). In this experiment, unlike the trend of effective spike numbers, all planting patterns exhibited a gradual decrease in the grains per spike and 1000-grain weight as the planting density increased, which is consistent with previous research findings.

Notably, the normal patterns (P1 and P3) showed larger declines in relation to the corresponding EBS patterns (P2 and P4) (compared to D1, when planting density increased to 630 × 10^4^ plants ha^−1^ (D2), the reductions in grains per spike for P1 and P3 were 6.6% and 4.5%, respectively; when increased to 690 × 10^4^ plants ha^−1^ (D3), the reductions were more pronounced at 14.1% and 12.0%, respectively; and when increased to 750 × 10^4^ plants ha^−1^ (D4), they further significantly decreased to 22.6% and 19.1%, respectively) (**Figure 2**). The EBS patterns (P2 and P4), however, were able to mitigate the reduction in grains per spike to some extent (compared to D1, when planting density increased to D2, the reductions in grains per spike for P2 and P4 were only 4.0% and 1.5%, respectively; when increased to D3, the reductions were only 5.2% and 5.6%, respectively; and when increased to D4, the reductions were further limited to 10.8% and 11.7%, respectively). Therefore, the grains per spike in EBS patterns (P2 and P4) were generally significantly higher than those in the corresponding normal patterns (P1 and P3) under high-density drip-irrigated systems (D2, D3, and D4). In this experiment, the change trend of 1000-grain weight was similar to that of grain number per spike, that is, compared with normal patterns (P1 and P3), EBS patterns (P2 and P4) could effectively mitigate the decreased degree of 1000-grain weight to a certain extent. Moreover, the 1000-grain weight of the EBS patterns (P2 and P4) was also significantly higher than that of the normal patterns (P1 and P3) under the high-density drip-irrigated system (D2, D3and D4) in most cases (**Figure 2**). As previously mentioned, the grain yields of the EBS patterns (P2 and P4) were generally higher than those of the corresponding normal patterns (P1 and P3) under high-density drip-irrigated systems (**Table 1**). It can be observed that the excellent performance of grain number per spike and 1000-grain weight of EBS patterns (P2 and P4) under high-density planting conditions in obviously presence parallel to the change trend of grain yield between planting patterns (**Figure 2**; **Table 1**). Therefore, we believe that the EBS patterns (P2 and P4) effectively mitigated the reductions in grains per spike and 1000-grain weight, making a significant contribution to maintaining grain yield under the high-density drip-irrigated wheat system.

### 3.3 Superior dry matter accumulation and redistribution ability of EBS planting patterns in wheat may play a crucial role in maintaining grain weight and grains per spike

**Figure 3:**
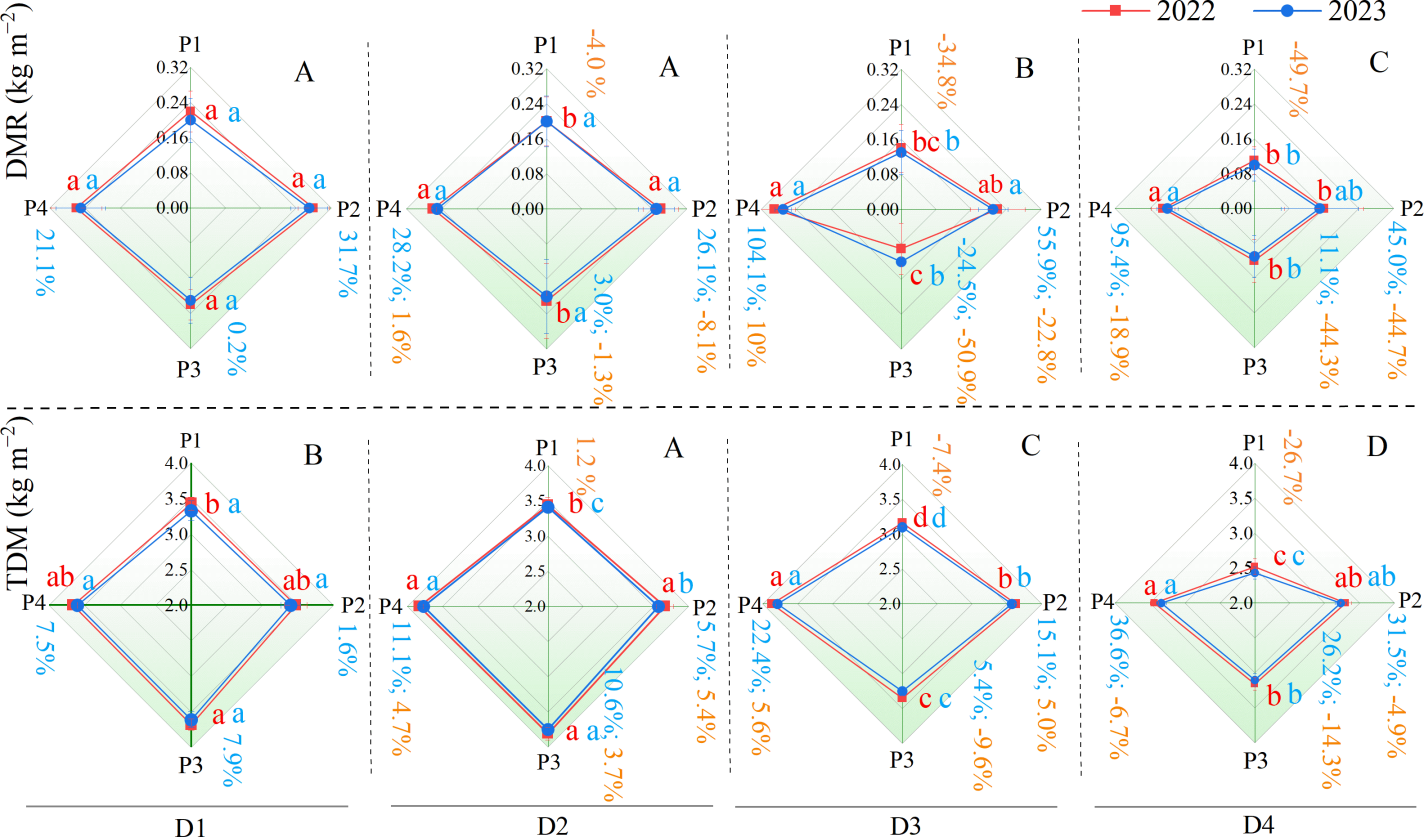
Performance of total dry matter accumulation (TDM), and dry matter remobilized after anthesis (DMR) for the four patterns. Lowercase letters indicate significant differences (*P* < 0.05) in wheat TDM and DMR among planting patterns at the same planting density level. Uppercase letters indicate significant differences (*P* < 0.05) in wheat TDM and DMR among different planting densities within the same planting pattern. Yellow numbers represent the percentage change in wheat TDM and DMR of each planting pattern under high-density planting levels (D2, D3, and D4) compared with the conventional density (D1). Blue numbers represent the percentage change in wheat TDM and DMR of P2, P3, and P4 compared with P1 under the same planting density level.

The accumulation and redistribution of dry matter is a complex and critical process that significantly influences yield components such as grain weight and grains per spike (Moll et al., 1994). Wheat converts solar energy into chemical energy through photosynthesis and stores it as dry matter, which directly affects its yield potential (Liu et al., 2016; Lv et al., 2019). In terms of total dry matter accumulation (TDM), under the traditional planting density (D1), TDM was similar across all planting patterns, except in 2022 when P3’s TDM was significantly higher than that of P1 (**Figure 3**). When the planting density increased to D2, TDM increased for all patterns (with increases of 1.2%, 5.4%, 3.7%, and 4.7% for P1, P2, P3, and P4, respectively). This is understandable because the quantity of wheat plants increases with planting density, leading to an overall increase in biomass. However, when planting density was further increased to D3 and D4, TDM for normal patterns (P1 and P3) showed a declining trend (P1 and P3 decreased by 7.4% and 9.6%, respectively, under D3 conditions, and further decreased by 26.7% and 14.3%, respectively, under D4 conditions) (**Figure 3**). This suggested that while moderate increases in planting density could enhance dry matter accumulation, excessively high densities may limit it, which is consistent with previous research (Liu et al., 2021; Shao et al., 2024). Interestingly, the EBS patterns (P2 and P4) mitigated this trend, at least under D3 conditions, where P2 and P4’s TDM still showed an increasing trend (increases of 5.0% and 5.6%, respectively) (**Figure 3**). The TDM for the EBS patterns (P2 and P4) was significantly higher than that for the corresponding normal patterns (P1 and P3) under high-density drip-irrigated systems. Thus, in this experiment, compared with the normal patterns (P1 and P3), the EBS patterns (P2 and P4) were more conducive to maintaining wheat dry matter accumulation under dense planting conditions.

**Figure 4:**
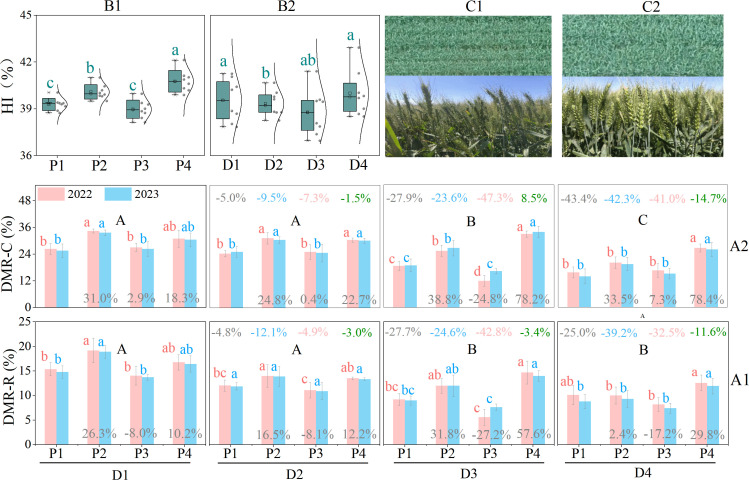
Performance of harvest index (HI), contribution rate of DMR to grains (DMR-C), and transfer rate of DMR to grains (DMR-R) of the four patterns. A1 and A2 represent the DMR-R and DMR-C performance of different drip irrigation spring wheat planting patterns under different planting densities, respectively. B1 and B2 indicate harvest index performance under different planting densities and planting patterns, respectively. C1 and C2 indicate wheat canopy uniformity and spike-layer neatness for the P2 and P4 planting patterns, respectively. Lowercase letters indicate significant differences (*P* < 0.05) in DMR-R and DMR-C among planting patterns at the same planting density. Uppercase letters indicate significant differences (*P* < 0.05) in DMR-R and DMR-C among different planting densities within the same planting pattern. Numbers at the top of each column represent the percentage change in DMR-R and DMR-C under high-density planting levels (D2, D3, and D4) compared with the conventional density (D1). Numbers at the bottom of each column represent the percentage change in DMR-R and DMR-C of P2, P3, and P4 compared with P1 at the same planting density. All percentage changes were calculated based on average data from the 2022 and 2023 growing seasons.

Previous research has demonstrated that competition among plants can intensify as the planting density increases (Shao et al., 2024). High-density planting often affects wheat nutrient distribution, prioritizing plant growth and development under limited nutrient conditions while allocating fewer nutrients to the grains (Liu et al., 2021). Our experimental data indicate that the amount of dry matter stored in nutrient organs before anthesis and then translocated to grains after anthesis (DMR) generally shows a gradual decreasing trend with increasing planting density (Liu et al., 2021; Shao et al., 2024). Similar to the TDM trend, in most cases, under dense planting conditions (D2, D3, and D4), the DMR of the EBS patterns P2 and P4 was significantly higher than that of the normal patterns (P1 and P3) at the same densities. This suggests that compared to normal patterns (P1 and P3), EBS patterns (P2 and P4) are more conducive to promoting the translocation of dry matter from nutrient organs to grains after anthesis under dense planting conditions. The transfer rate of DMR to grains (DMR-R) and contribution rate of DMR to grains (DMR-C) further supported this conclusion (**Figure 4**). Overall, both the DMR-R and DMR-C of wheat in all planting patterns decrease with increasing planting density (with reductions in DMR-R ranging from 4.8% to 25.0%, 12.1% to 39.2%, 4.9% to 42.8%, and 3.0% to 11.6% for P1, P2, P3, and P4, respectively, under high-density planting conditions (D2, D3, and D4), and reductions in DMR-C from 5.0% to 43.4%, 9.5% to 42.3%, 7.3% to 47.3%, and 1.5% to 14.7%, respectively) (**Figures 4A1, A2**). It is evident that the EBS patterns of P2 and P4 can significantly mitigate the reductions in DMR-R and DMR-C under dense planting conditions (in most cases, the EBS patterns P2 and P4 were also significantly higher than the normal patterns of P1 and P3 at the same density level). This ultimately enhanced the economic yield (grain yield) of the EBS patterns (P2 and P4), leading to a significant improvement in the harvest index (**Figure 4B1**).

In fact, increasing planting density can compact the canopy structure and increase the photosynthetic surface area, but it may also limit light penetration through the crop, restrict photosynthesis and ventilation, and thereby inhibit the translocation of photosynthates to grains (Gao et al., 2025; Liu et al., 2021). Our experimental results indicated that while increasing planting density enhanced the number of effective spikes, DMR, DMR-R, and DMR-C significantly decreased with higher density, thereby weakening grain yield formation and ultimately leading to a downward trend in the Harvest Index (HI) (**Figure 4B2**), consistent with previous studies (Liu et al., 2021). However, when the planting density was increased to (D4), HI showed an upward trend. This may be related to a significant reduction in biomass (total above-ground biomass), such as thinner plant stems and increased lodging. Notably, the performance of dry matter accumulation and transport (TDM, DMR-R, and DMR-C) for the EBS uniform sowing pattern (P4) outperformed the EBS drilling sowing pattern (P2) (**Figures 3**, **4**). This is understandable, as the uniform sowing pattern allows for more uniform growth space for wheat, potentially improving the population structure by enhancing light penetration and photosynthetic rates in the middle and lower layers of the canopy, thus promoting biomass accumulation and transport capacity. In contrast, the distribution of light penetration in drilling sowing wheat might be uneven, thereby limiting photosynthesis in the middle and lower leaves. From the perspective of the wheat canopy, the uniformity and neatness of the canopy in P4 (**Figure 4C2**) were clearly superior to those in P2 (**Figure 4C1**). This further validated the mechanisms discussed above.

### 3.4 The border effects played a significant role in optimizing the dry matter accumulation and remobilization of EBS patterns

**Figure 5:**
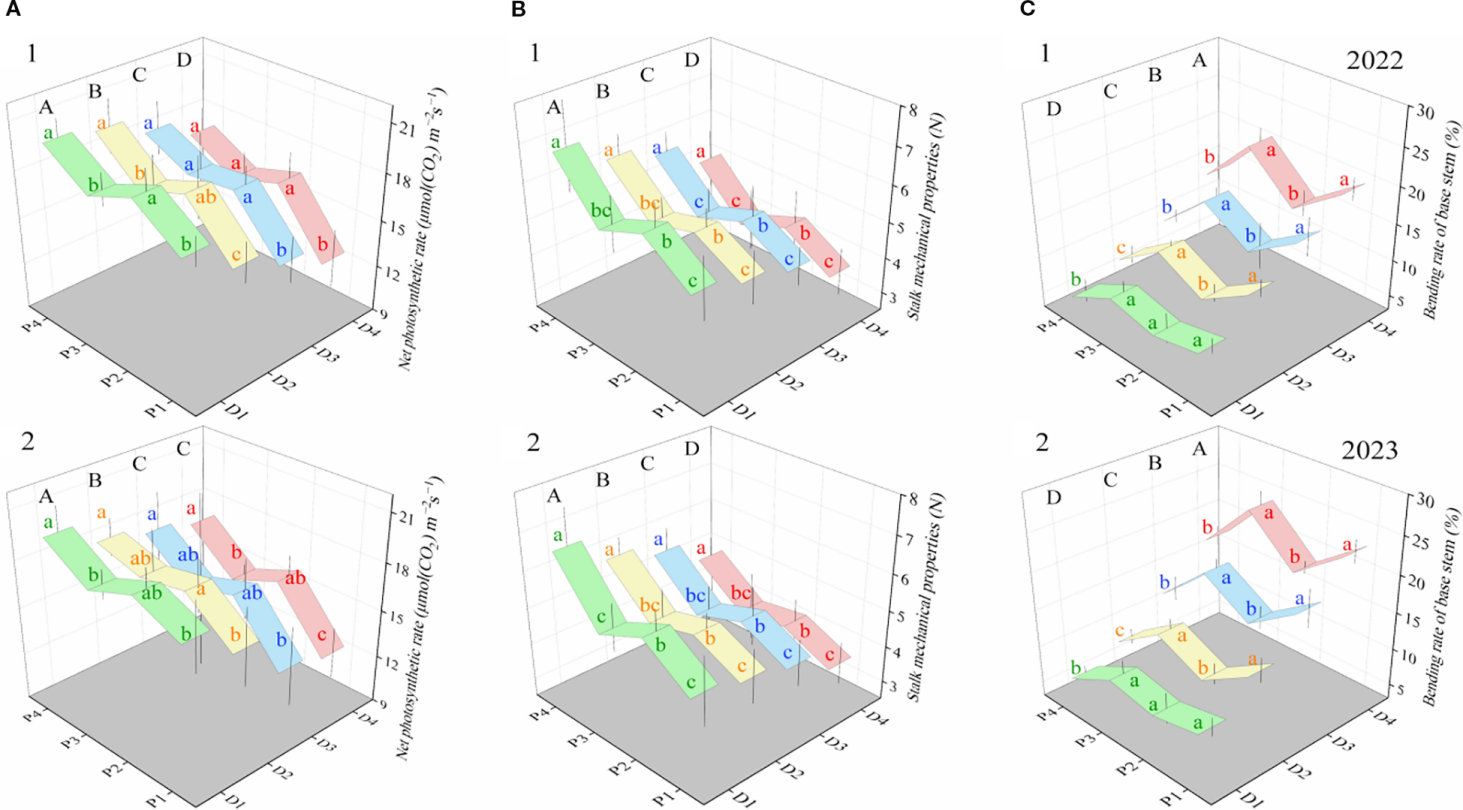
Performance of the net photosynthesis rate of wheat flag leaf, mechanical strength of wheat stalk, and bending rate of wheat base stem of the four patterns. **(A1, A2)** represent the net photosynthetic rates of flag leaves under different drip irrigation spring wheat planting patterns and planting densities, respectively. **(B1, B2)** indicate the mechanical strength of wheat stalks under different planting patterns and planting densities, respectively. **(C1, C2)** indicate basal stem bending rates under different planting patterns and planting densities, respectively. Lowercase letters indicate significant differences (*P* < 0.05) among planting patterns at the same planting density level for each trait. Uppercase letters indicate significant differences (*P* < 0.05) among planting densities within the same planting pattern.

Photosynthesis is one of the most important physiological processes during the growth and development of wheat, providing not only an energy source but also directly affecting biomass accumulation and material translocation (Lyu et al., 2021; Wan et al., 2023; Chen et al., 2024). Therefore, the differences in biomass accumulation and redistribution among different planting patterns should first be explained from the perspective of photosynthetic performance. Studies have shown that as planting density increases, the net photosynthesis rate of wheat flag leaves tends to significantly decrease in most cases (only except in 2023, where D3 and D4 showed no significant differences) (**Figures 5A1, A2**). This trend parallels the decrease in wheat DMR, DMR-R, and DMR-C with increasing density (**Figures 3** and **4**). This further demonstrates that while increasing planting density can enhance the number of flag leaves in the wheat canopy, the reduction in net photosynthetic capacity ultimately hinders dry matter accumulation and redistribution to grains, thereby suppressing grain yield formation. The reduction in the net photosynthesis rate caused by increased density can be explained from two perspectives. First, because water and nutrient conditions are consistent, an increase in density reduces the nutrients and water available to each plant, thereby suppressing stomatal opening and the activity of photosynthetic enzymes, ultimately reducing photosynthetic performance (Xiang et al., 2016). Second, because increased density indeed intensifies leaf overlap and shading, the actual light radiation received by leaves may decrease, negatively affecting photosynthetic performance (Casal, 2013). Similar to dry matter accumulation and transport, the net photosynthesis rates in the EBS planting patterns (P2 and P4) were generally significantly higher than those in the normal patterns (P1 and P3). Previous studies have shown that under the drip-irrigated system, the widening of the border row space can have a potential border effect, which usually makes the growth of the border-row crop plants more robust, thus conducive to improving the resilience ability of the whole planting system (Shen et al., 2023; Wan et al., 2023). In this experiment, expanding the border space to 20 cm was undoubtedly more advantageous for ventilation, aeration, and light penetration, potentially exploiting the marginal row effect to improve leaf photosynthetic performance and promote biomass accumulation and transport. Moreover, EBS not only enhances photosynthetic performance but also has a positive effect on the mechanical strength of wheat stems at the base. In fact, in our previous experiments, we had conducted in-depth research on the mechanism by which EBS can effectively enhance the lodging resistance of drip-irrigated wheat using the path analysis method. EBS can usually optimize the growth state of the flag leaves (the angle and height of the flag leaves) of wheat, promote the accumulation of endogenous substances (lignin and cellulose) in the second internode at the base of the stem, thereby effectively increasing the substantial degree and mechanical strength of the stem and exerting the lodging resistance effect (Wan et al., 2023). The experimental data confirm that the aforementioned mechanism remains effective even under high planting density conditions, i.e., the data indicate that although increased density significantly reduced the mechanical strength of wheat stems, compared to the normal patterns (P1 and P3), the EBS patterns (P2 and P4) generally improved stem strength under dense conditions (with stem strength in P2 and P4 generally being significantly higher than in P1 and P3) (**Figures 5B1, B2**). Due to the lodging of drip irrigation spring wheat mainly consists of stalk lodging at grain-filling stage and stalk lodging occurs most frequently at the basal second internode above the soil surface in Xinjiang (Wan et al., 2023). So enhance stem strength can further positively affect wheat lodging resistance (Wan et al., 2023). The bending rate of the wheat base stem in the EBS patterns (P2 and P4) was significantly lower than that of the normal patterns (P1 and P3) (only except where the rate of base stem bending between P2 and P1 showed no significant difference at D1) (**Figures 5C1, C2**). Because stems serve as vital conduits connecting wheat leaves and spikers, bending can obstruct the transport of dry matter from the stem phloem to the spike (Sterling et al., 2003; Berry et al., 2006). In particular, during grain filling, when the carbohydrates stored in the stem are rapidly transferred to the grains, stem bending may hinder dry matter transport, thereby affecting the rate of dry matter translocation to the grains (Sun et al., 2022). This is another important reason that explains why the conventional planting patterns (P1 and P3) have significantly lower dry matter accumulation and transport efficiency (**Figures 3** and **4**), as well as lower number of grains per spike and grain weight per spike (**Figure 2**) compared to the corresponding EBS patterns (P2 and P4) at high planting densities. Therefore, we believe that the relatively superior biomass accumulation and redistribution mechanisms in P2 and P4 are mainly related to border effects by expanding the border space under the drip-irrigated spring wheat system.

### 3.5 Uniform canopy structure and consistent grain maturity further enhance the yield-increasing effect of the EBS uniform sowing pattern (P4)

**Figure 6:**
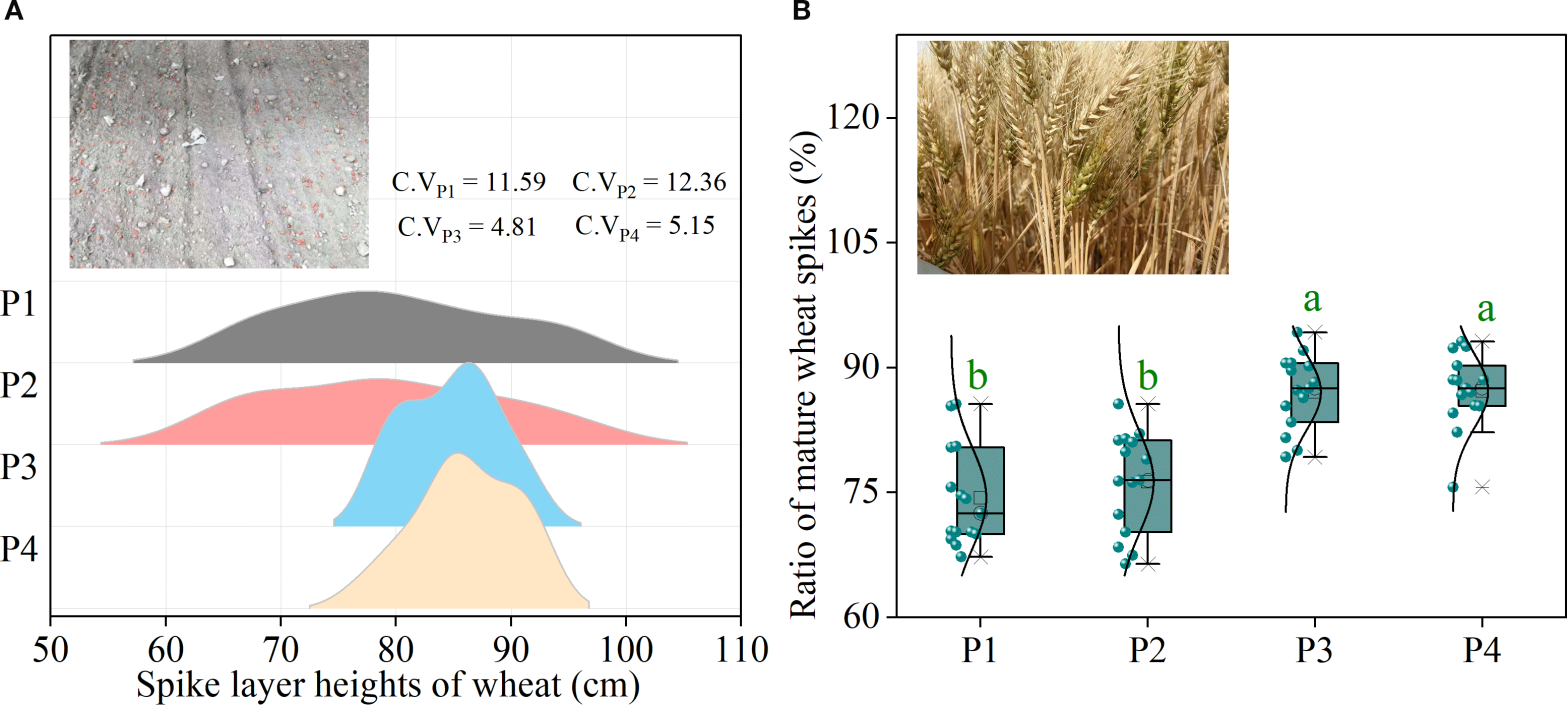
Performance of wheat spike layer heights and mature wheat spike ratio under the four patterns. **(A)** indicates the spike layer height under different drip irrigation spring wheat planting patterns. **(B)** indicates the ratio of mature wheat spikes under different drip irrigation spring wheat planting patterns. Lowercase letters indicate significant differences (*P* < 0.05) among planting patterns at the same planting density level. C.V_P1_, C.V_P2_, C.V_P3_ and C.V_P4_ represent the coefficients of variation in the spike layer height under the respective planting patterns.

To improve sowing quality, a precision wheat sowing planter was employed in this experiment to address the limitations of previous studies that used manual sowing methods for uniform sowing pattern research. This ensured the maximum seed distribution uniformity (**Supplementary Figure S3**). Previous research indicates that when wheat seeds are uniformly distributed in the field, each wheat plant can access equal growth space and resources (such as sunlight, water, and nutrients), which is conducive to the uniform growth of wheat plants, thereby reducing the inconsistency in wheat spike layer height caused by differences in the growth environment (Wang, 2024). When the wheat spike layer height is uniform, wheat plants have a relatively equal growth environment and resource allocation during the growth process (Li and Wei, 2021; Wang, 2024). This can help promote synchronized growth and development of wheat spikes, enabling them to reach the mature state at a similar time (with similar grain-filling rates and ripening stages) (Shang and Zhang, 2023). The experimental data showed that wheat with uniform sowing patterns (P3 and P4) exhibited a more stable spike layer height, and the coefficient of variation (C.V) for spike layer height in the uniform sowing patterns (C.V range: 4.81–5.15) was significantly lower than that in the row sowing patterns (P1 and P2) (C.V range: 11.59–12.36) (**Figure 6A**). A more stable canopy allows the spike layer to receive uniform solar radiation and promotes more wheat spikes to mature at the same time (Li and Wei, 2021). The data also indicated that the ratio of mature wheat spikes (defined as those with a grain moisture content less than 13%) was significantly higher in the uniform sowing patterns (P3 and P4) at the maturity stage than in the row sowing patterns (P1 and P2) (**Figure 6B**). A higher ratio of mature spikes suggested that the uniform sowing patterns promoted more spikes to undergo complete grain filling, which was beneficial for increasing total grain weight. This essentially explains why the yield and biomass accumulation in the EBS uniform sowing pattern (P4) generally outperformed those in the EBS row sowing pattern (P2) (**Figure 4C2** and **Table 1**). Moreover, because of the arid climate in Xinjiang (where the annual evaporation (approximately 2000 mm) is more than ten times the rainfall (about 200 mm)), locally harvested wheat is typically sold or stored directly without sun-drying (Lv et al., 2019; Wan et al., 2022a). Therefore, in actual production, to ensure the safe storage of wheat grains, large-scale mechanical harvesting is typically delayed until immature spikes (in this experiment, spikes with grain moisture content exceeding 13% were classified as immature, particularly wheat spikes from stunted plants) have fully completed grain filling (Niu et al., 2024). Nevertheless, this waiting process inevitably causes a large amount of grain shedding from the initially matured spikes, thus resulting in serious yield losses. In contrast, uniform sowing patterns (P3 and P4) promoted more synchronized wheat spike maturation within the same timeframe. This mechanism may be conducive to centralized harvesting of wheat for 3–7 days in advance. While reducing the loss of grain threshing, it creates a longer growth duration for multiple cropping crops (such as after wheat harvest, multiple cropping maize and soybeans in northern Xinjiang) and improves agricultural production efficiency. Thus, given the superior performance in yield, WUE, economic benefits, and other factors, we prioritize recommending the EBS uniform sowing pattern for promotion under drip irrigation dense planting conditions in Xinjiang to achieve synergy in stabilizing and increasing grain production, further ensuring food security.

## 4 Conclusion

We explores the effects of expanding border space (EBS) within various drip irrigation patterns on yield performance, water use efficiency, and lodging resistance in high-density spring wheat cultivation in Xinjiang. The experimental results showed that the EBS patterns (P2 and P4) exhibited better tolerance to high planting densities. The reasons were (1) EBS can effectively mitigate the decrease degree of grain weight and grain number per spike under the condition of dense planting. (2) EBS can improve the ability of dry matter pre-stored in vegetative organs before anthesis, which is subsequently redistributed into grains during grain filling, thereby increasing the harvest index. (3) EBS can have a potential border effect, improve photosynthetic performance, and reduce the risk of stem bending, thereby improving lodging resistance. Additionally, uniform sowing promoted synchronous development of wheat spikes and reduced harvest losses in relation to drilling sowing. Overall, the EBS pattern of P4 was recommended for high-density drip-irrigated spring wheat systems because of its superior yield performance, stability, water use efficiency, and economic benefits. The findings of this experimental study are applicable to other arid and semi-arid regions.

## Data Availability

The original contributions presented in the study are included in the article/**Supplementary Material**. Further inquiries can be directed to the corresponding author.

## Supplementary material

Supplementary Figure 1 Diagrams of the drip irrigation patterns. TR4, TR6, TR6L, TR6S, TR8, TR8L and TR8S indicate one tube serves four rows of wheat plants with common row space of 15 cm (control), one tube serves six rows of wheat plants with common row space of 15 cm, one tube serves six rows of wheat plants with narrow row space of 10 cm and inter-block space of 35 cm, one tube serves six rows of wheat plants with row space of narrow 10 cm and inter-block space of 25 cm, one tube serves eight rows of wheat plants with common row space of 15 cm, one tube serves eight rows of wheat plants with narrow row space of 10 cm and inter-block space of 45 cm, one tube serves eight rows of wheat plants with row space of narrow 10 cm and inter-block space of 25 cm, respectively.

Supplementary Figure 2 The pre-experimental wheat canopy imaging under dense planting.

Supplementary Figure 3 The seeding effect of uniform sowing planters.
