# Supplementary material for: Expanding border space improve both yield and stability in a high-density drip-irrigated spring wheat system in Xinjiang, China
Source: Front Plant Sci. 2025 Oct 21;16:1655063. doi: 10.3389/fpls.2025.1655063 (PMC12584056; doi:10.3389/fpls.2025.1655063)
Supplement: Supplementary Figure 1 — Diagrams of the drip irrigation patterns. TR4, TR6, TR6L, TR6S, TR8, TR8L and TR8S indicate one tube serves four rows of wheat plants with common row space of 15 cm (control), one tube serves six rows of wheat plants with common row space of 15 cm, one tube serves six rows of wheat plants with narrow row space of 10 cm and inter-block space of 35 cm, one tube serves six rows of wheat plants with row space of narrow 10 cm and inter-block space of 25 cm, one tube serves eight rows of wheat plants with common row space of 15 cm, one tube serves eight rows of wheat plants with narrow row space of 10 cm and inter-block space of 45 cm, one tube serves eight rows of wheat plants with row space of narrow 10 cm and inter-block space of 25 cm, respectively. [file Supplementaryfile1.pdf]

Supplementary Fig. 1 Diagrams of the drip-irrigated patterns

Notes: TR4, TR6, TR6L, TR6S, TR8, TR8L and TR8S indicate one tube serves four rows of wheat plants with common row space of 15 cm (control), one tube serves six rows of wheat plants with common row space of 15 cm, one tube serves six rows of wheat plants with narrow row space of 10 cm and inter-block space of 35 cm, one tube serves six rows of wheat plants with row space of narrow 10 cm and inter-block space of 25 cm, one tube serves eight rows of wheat plants with common row space of 15 cm, one tube serves eight rows of wheat plants with narrow row space of 10 cm and inter-block space of 45 cm, one tube serves eight rows of wheat plants with row space of narrow 10 cm and inter-block space of 25 cm, respectively.

Supplementary Fig. 2 The pre-experimental wheat canopy imaging under dense planting

Supplementary Fig. 3 The seeding effect of uniform sowing planters

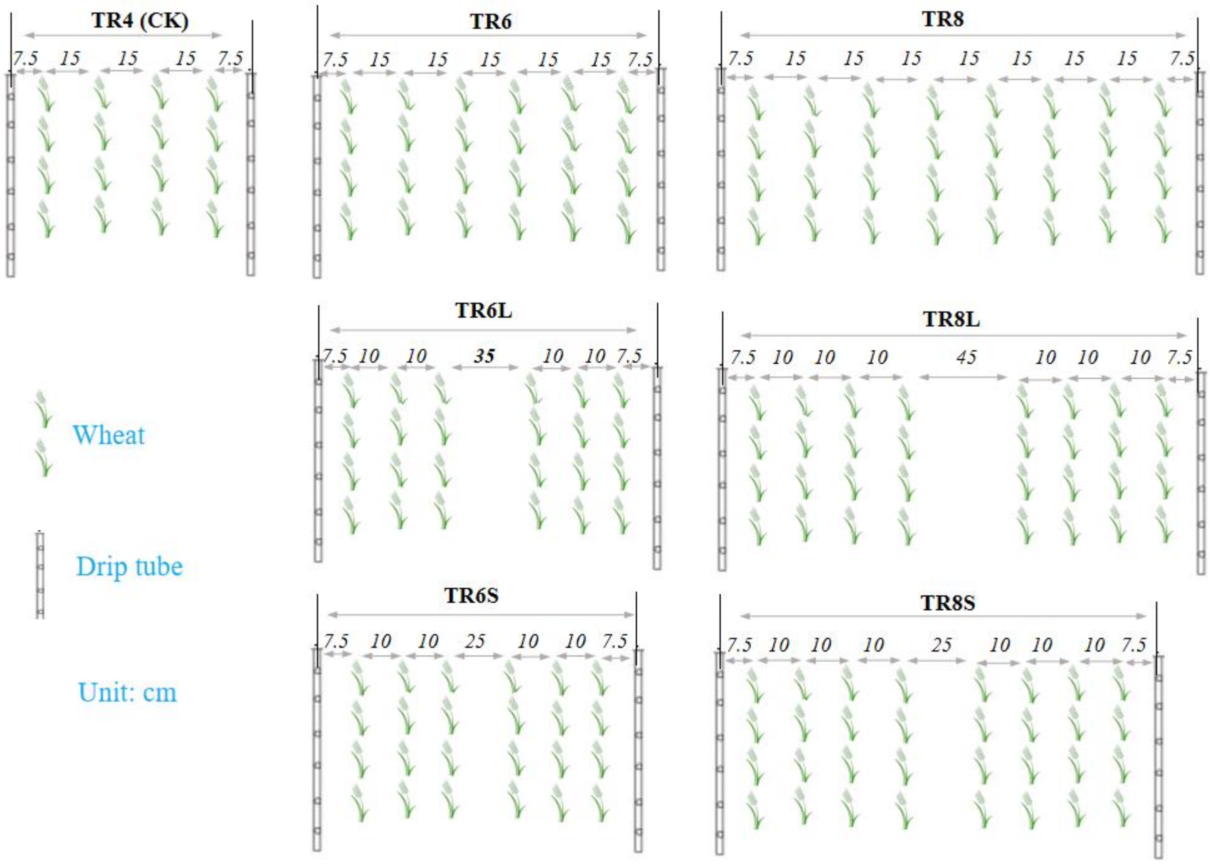

Supplementary Fig. 1

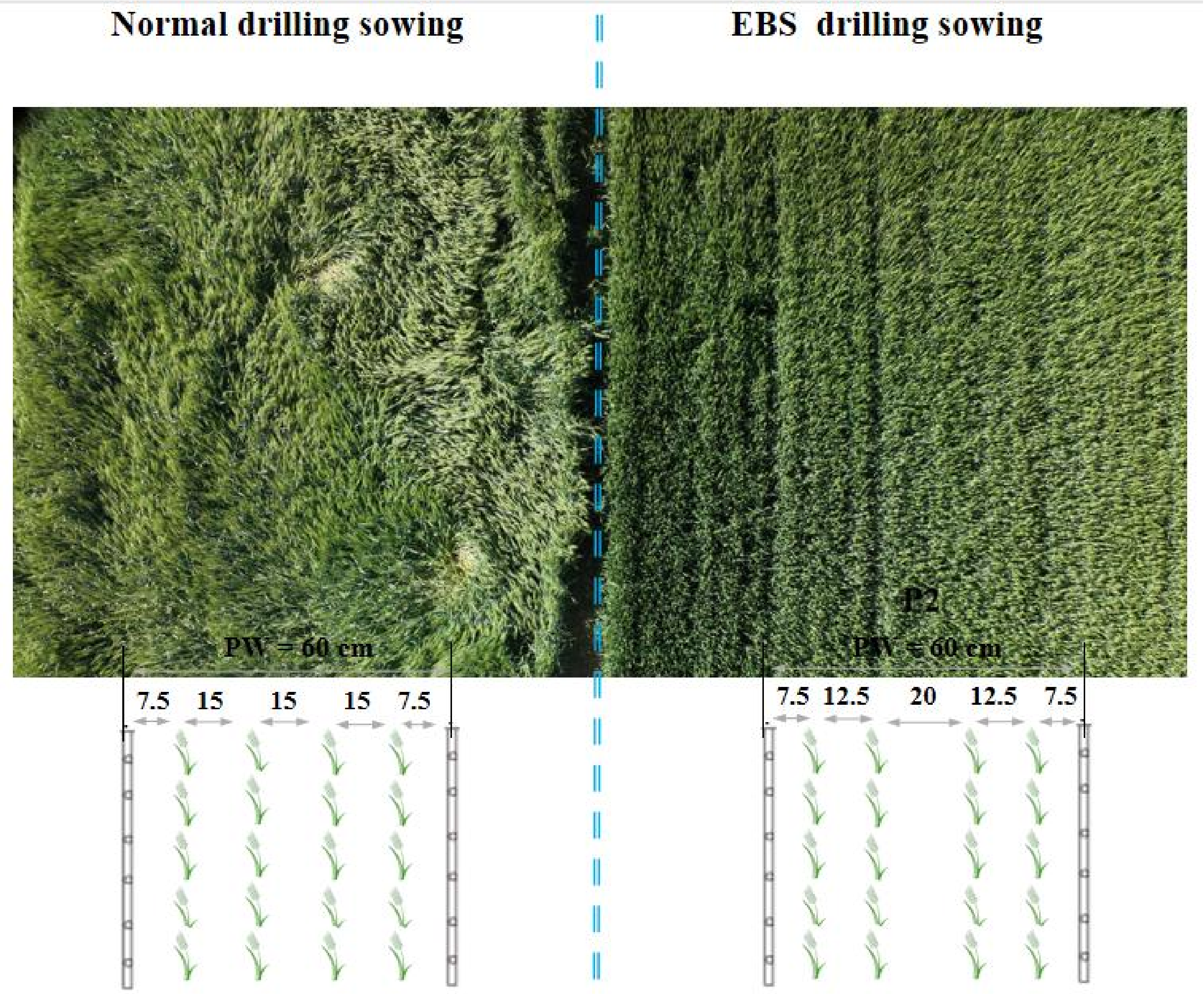

Supplementary Fig. 2

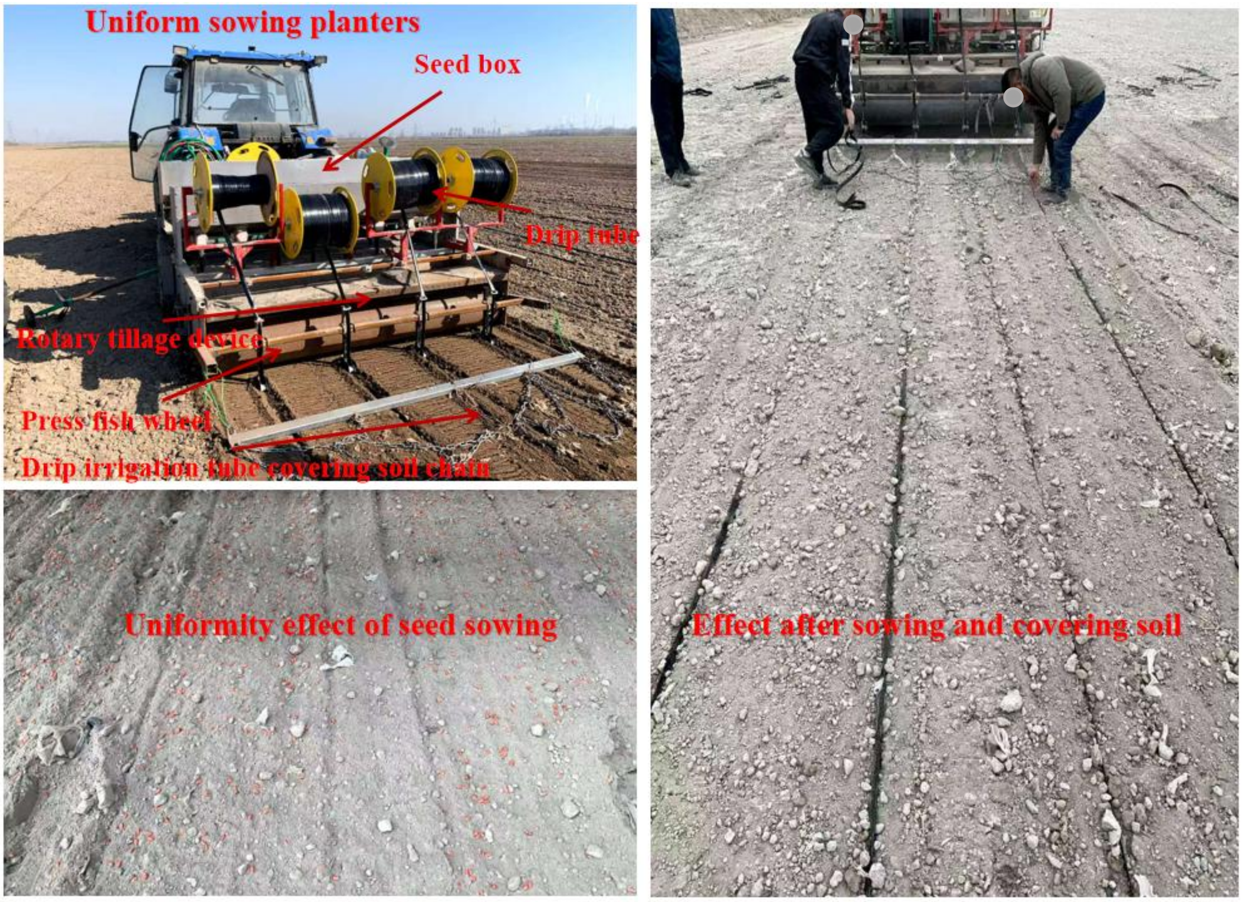

Supplementary Fig. 3
